# Developing a mobile application to better inform patients and enable effective consultation in implant dentistry

**DOI:** 10.1016/j.csbj.2016.06.006

**Published:** 2016-06-29

**Authors:** Erokan Canbazoglu, Yucel Batu Salman, Mustafa Eren Yildirim, Burak Merdenyan, Ibrahim Furkan Ince

**Affiliations:** a Vocational School of Technical Sciences, Akdeniz University, 07058, Antalya, Turkey; b Software Engineering Dept., Bahcesehir University, 34353 Besiktas, Istanbul, Turkey; c Electrical and Electronics Engineering Dept., Bahcesehir University, Besiktas, Istanbul, Turkey; d Department of Computer Science, University of York, United Kingdom; e Computer Engineering Department, Gediz University, Izmir, Turkey

**Keywords:** Implant procedures, Patient education, Interface design, Dental applications, Mobile devices

## Abstract

The field of dentistry lacks satisfactory tools to help visualize planned procedures and their potential results to patients. Dentists struggle to provide an effective image in their patient's mind of the end results of the planned treatment only through verbal explanations. Thus, verbal explanations alone often cannot adequately help the patients make a treatment decision. Inadequate attempts are frequently made by dentists to sketch the procedure for the patient in an effort to depict the treatment. These attempts however require an artistic ability not all dentists have. Real case photographs are sometimes of help in explaining and illustrating treatments. However, particularly in implant cases, real case photographs are often ineffective and inadequate. The purpose of this study is to develop a mobile application with an effective user interface design to support the dentist–patient interaction by providing the patient with illustrative descriptions of the procedures and the end result. Sketching, paper prototyping, and wire framing were carried out with the actual user's participation. Hard and soft dental tissues were modeled using three dimensional (3D) modeling programs and real cases. The application enhances the presentation to the patients of potential implants and implant supported prosthetic treatments with rich 3D illustrative content. The application was evaluated in terms of perceived ease of use and perceived usefulness through an online survey. The application helps improve the information sharing behavior of dentists to enhance the patients' right to make informed decisions. The paper clearly demonstrates the relevance of interactive communication technologies for dentist–patient communication.

## Highlights

• Presents a case study using user-centered development methodology
• The quality of doctor patient interaction is an important driver of satisfaction.
• Several patients are annoyed with bloody photos, particularly in implant cases.
• Rich visual content by mobile devices has positive effect on user experience.
• Actual users' participation into design is effective to reduce system complexity.

## 1 Introduction

The interaction between dentists and patients has become an important concern in making appropriate treatment decisions [1], [2]. The primary components of dentist–patient communication are information exchange, treatment decision and interpersonal relations [3]. In order to make a favorable decision towards treatment it is vital that the patient clearly understands the treatment plan [4], [5]. One of the important ways of gaining patients' trust and providing the patient with a sense of security is through effective patient-centered communication [6]. However, due to dentists' scheduled workload this crucial information exchange process can be overlooked and undervalued. In such cases, the information needs to be transferred fast and effectively to ensure that the patient has gained full understanding of the procedures. Improving communication skills along with a display of sympathy to the patient in a professional manner by a dentist can certainly make the patient feel more at ease and relaxed [7].

Dentists experience difficulties in explaining the treatment plan to their patients only through verbal or pictorial presentations. Often dentists make desperate attempts, through sketching, to present the procedure to the patient, who is frequently unfamiliar with the subject. These attempts however, require an artistic ability that is not a precondition of becoming a dentist. Real case photographs are sometimes helpful to explain and illustrate procedures. However, particularly in implant cases, real case photographs are frequently ineffective and inadequate. Effective dentist–patient communication cannot be established merely based on using paper-based presentations.

Briskly progressing technological improvements have allowed for more powerful, graphics-oriented applications to be created and utilized for patient presentations. The decision to develop an application compatible with tablet devices was made because of their portability and high performance. Currently, these devices can perform anything a standard PC can, just in a more portable format. These portable tablet devices eliminate the need for a standing PC in every room and allow dentists to gain flexibility. An efficient communication environment, supported by interactive systems with visual presentations should be implemented for better explanation of further treatment plans. Information technology developed for dentistry is relatively more limited than systems available for the wider medical industry. Thus, there is a need for a mobile application with rich graphical content to help inform patients about alternative methods of treatment, reduce confusion, improve the service quality, and enable correct use of medication [8].

The last three years have been marked by rapid adoption of mobile devices. In the United States, researchers predicted that 20% of the populations use touchscreens with natural user interfaces as their primary computing device by 2014 [9]. 37% of the populations own such devices for business purposes [10]. In December 2014 there were 49 million mobile subscribers supported with 3G connections in Turkey [11]. 945,254 tablet computers were purchased by Turkish users in the fourth quarter of 2014 [11]. The number of tablets sold in Western Europe is to be 47.6 million units in 2015 [12]. Approximately 83.7 million tablet pcs were sold in the USA in 2015 [12].

In this study, we designed a mobile application to be used to inform patients about treatment plans in office settings. The application was developed for dentists by implementing user centered development methodology followed by testing. User centered approaches are beneficial in gaining insights in the healthcare domain, and for identifying the knowledge and requirements of all stakeholders [13]. The integration of potential users in the software lifecycle reduces the number of iterative developments and the users' training costs [14]. Physicians believe that developers should be more interested in their preferences, and visit their practice environment in order to clearly identify the context. The collaboration between the development team and the dentists is improved by implementing this methodology [15]. Salman et al. proposed icon design guidelines and designed medical icons for a mobile emergency service application. It was found that the participatory icon design guidelines resulted in usable and clear icons, which improved the system usability and user success [16].

Soft and hard tissues were modeled in 3D computer aided design programs and the most frequently encountered implant cases were prepared for both dentists and patients. The system enables the presentation of implant supported prosthetic treatments to the patients with 3D rich illustrative content. It enables dentists to easily explain treatment plans to their patients when there is a need to describe surgical operations.

The treatment decision making should be managed together and the patient has the right to be informed in details. The portability of new generation devices provides flexibility to dentists in effectively communicating with the patients. The contribution of this research is in the designing of the application with the participation of actual users, implementing user-centered design methodology to remove the communication barriers between dentists and patients. Few researches focused on computerized systems in a dental environment, and these papers do no present a mobile application. However, our application was developed specifically for implant procedures. Additionally, we found encouraging outcome as dentists indicated that they are willing to continue using this application in the future.

### 1.1 Scientific background

Wingard describes patient education as the process of informing the patients by health professionals in altering patients' health behaviors, improving health status, and aiding in development of remedy treatment [17]. The aim of patient education is to assure that the patients are informed of their treatment options, efficient use of medication, and the management of their healthcare needs [18]. It is also critical for improving self-advocacy in deciding to act independently from medical provider and increasing patient motivation.

In addition to the complexities involved with developing education programs that meet the needs of a highly diverse population, resources available to healthcare providers have come under significant financial pressures. This challenging environment has made it essential that healthcare providers take a more systematic, coordinated, and strategically planned approach that would facilitate the effective deployment of the resources allocated to patient education [19].

Mobile systems developed to support the interaction between physicians and patients are now replacing paper based methods [20]. The expanding use of portable devices in the education of physicians and students is a well-documented phenomenon [21]. Tablet devices have become useful for decision making purposes and are increasingly used in clinical settings as a reference tool [22]. The use of mobile applications has emerged as an educational method that may assist dentists in meeting financial and strategic challenges. These applications may provide a highly cost effective modality for delivering content [23]. Tablet devices have various multimedia capabilities such as CT images and drawing tools that are useful in increasing clinical efficacy, improving the patient experience, and optimizing patient satisfaction [24], [25].

Various studies measure the effect of interactive education applications on users' overall satisfaction with their healthcare encounter [26], [27], [28]. The quality of patient education may also affect patients' health conditions. While some studies reported no benefits of using such applications in this context [29], [30], [31], [32], [33], others reported a positive impact of the use of interactive systems [34], [35], [36], [37], [38], [39], [40], [28].

It is suggested that physicians' job satisfaction is a perception based reaction that results from a number of variables including the nature of relationships with patients [41], [42], [43]. The quality of doctor–patient interactions is consistently noted as an important driver of satisfaction [42]. Haas et al. found that more satisfied physicians are better communicators and more empathetic [43]. In turn, patients with higher satisfaction prompted physicians to feel better about the care they provided, which motivated physicians to spend more time meeting patients' needs. Physicians also believe that there is a brilliant future at hand where several clinical practices, such as lab work and CT images are carried out using a mobile device [44].

### 1.2 Rationale for the study

The portability of tablet devices provides efficient communication with patients not only in clinical settings, but also in eliminating the location barriers. To enhance patient education dentists need to be able to illustrate the benefits of treatment easily, show the consequences of untreated cases, and demonstrate dental concepts from simple fillings to complex implant procedures [45], [46].

**Fig. 1:**
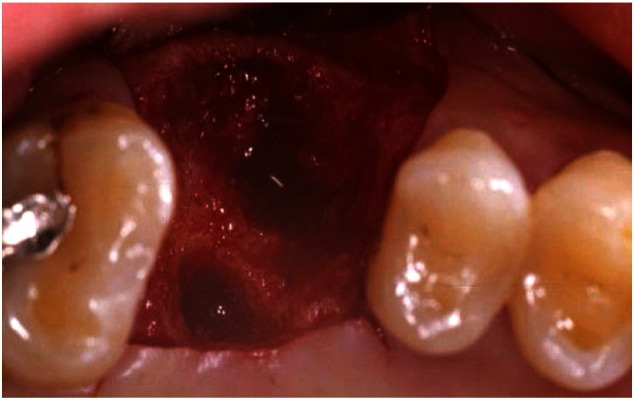
Real case photograph.

The primary function of our application is to inform patients by using the new generation of hardware and communication technologies in implant dentistry to support treatment decision making. The treatment plan is better explained to patients using 3D illustrations through a mobile device, which also helps improve the service quality in terms of time and effort spent, and enables better understanding of treatments. It was observed that prior to using our application our dentists were using real images from a book, and sketches, which have the potential to cause frustration and fear in the patients (Fig. 1). We witnessed several failed conversations between dentists and patients during our on-site visits.

It was crucial to get a quick understanding of the content preferences of the dentists in creating innovative ideas for our application. At the very beginning of the research, an online survey was given to potential users in order to identify the potential areas of implementation for our application. The aim was to identify the primary demands of dentists regarding the content to be included in the system. Sampling was used to cover as wide a population of dentists as possible at the early stages in the lifecycle process. 1610 dentists participated in the online survey through an official dentistry web portal (www.dis-hekimi.org) which provides up-to-date information on treatment methods for various dental cases. Dentists attended to this initial survey are all Turkish located at several cities of Turkey.

**Fig. 2:**
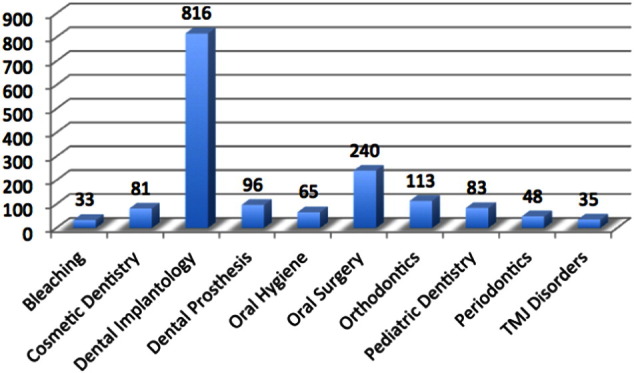
The results of the initial survey.

32% of participating dentists own tablet devices. Among those surveyed, implant dentistry was chosen as the must-have content by 51% of participating dentists. Oral surgery, orthodontics, and cosmetic dentistry were selected by 15%, 6%, and 5% respectively. Fig. 2 shows the results of the initial survey.

A dental implant is an artificial tooth root which is placed into the jaw to hold a prosthetic replacing a damaged or missing tooth. The implant includes a crown, an abutment, and an implant body. The global dental implant and prosthetics market was valued at US$6.8 billion dollars in 2011 and is expected to increase to US$ 10.5 billion dollars in 2016 [47]. It is crucial to satisfy the needs of patients in the treatment process through the integration of technological advances.

### 1.3 Research objectives

The main goal of this study is to improve the dentist–patient interaction and patient education by implementing a mobile application in the dental context. It is assumed that this application will support patient–dentist communication and might improve the satisfaction of both parties by providing better interaction. The suitability of user centered design methodologies, which involve the actual users in each phase of the software development lifecycle, was also tested. This study presents the relevance of designing a mobile application using interactive communication technologies for supporting patient education and communication.

## 2 Design and development

Our design methods include task and user analysis, user interface design, and evaluation with the actual users' participation. This research is primarily conducted to cover the design process of the application. Task analysis was conducted by interviewing and observing at the work site. Sketching, paper prototyping, and wireframing stages were completed for the initial design. Apple Human Interface Guidelines (HIG) principles were taken into consideration while designing the user interfaces [48]. These guidelines are established to help users to understand and interact with the content. User interface concepts for providing improved interaction, such as layout, navigation, animation, color, typology, icons, graphics, and terminology use for tablet devices are described in detail [48]. Superlative user interfaces and user experiences were achieved for the application by using good platform practices.

Displaying relatively large content on a tablet device was the primary challenge in this study. The most efficient hierarchical menu system was investigated for fast processing. The solutions were generated to organize the user interface elements and 3D illustrations for the benefit of dentists. Finally, in-screen and platform-specific prototypes for the application were developed.

An online survey was conducted in the evaluation stage with the participation of 100 dentists. The subjects were briefly informed about the purpose of the study and allowed to use the application for two weeks in their natural settings. The participants were kept updated and encouraged to use our application for their own dental workflow during testing. Two commonly used measures; perceived ease of use (PEU), and perceived usefulness (PU) were inspected [49].

### 2.1 Application development methodology

User-centered development methodology includes actual users through the analysis, planning, design and development of a specific product [50]. It includes five steps: (1) understand the context of use; (2) specify the user requirements; (3) generate design solutions to meet the user requirements; (4) evaluate designs against requirements; and (5) check whether design solutions satisfy user requirements [50]. User characteristics such as experience, knowledge, education, preferences, and habits should be identified. The primary purpose of including end-users is to ensure that the final product is usable and meets the users' needs [51].

In the predesign stage, both ethnographical observations and contextual inquiry techniques were used. Cognitive walkthrough was used to identify work breakdown structure in the early design stage. We developed the system using a four step method: (1) determine the users and usage context, (2) identify the functional and business requirements, (3) design the system from rough concepts, and (4) evaluation.

### 2.2 User and task analysis

Five private dental clinics in Istanbul, Turkey were visited in order to observe real cases and to understand the medical environment. Initially, to better figure out the existing medical environment, several context-related cases were observed without interacting with on-site staff. Additionally, dentists were interviewed to identify the treatment and medical procedures to be included to the system. The accuracy of each scenario and corresponding tasks was validated by the interviewed dentists.

### 2.3 User interface design

A workshop was organized with 11 (3 female, 8 male) dentists. The average age of the subjects was 35. All subjects declared their familiarity with the technological developments and mobile devices. The workshop was conducted in a controlled environment, a standard computer laboratory at Bahcesehir University, Istanbul. The sessions took 2 days, 3 h/day and were recorded using a handy cam, and several photos were taken. Initially, some brief information about the experiment was provided to the subjects. The participants were encouraged to discuss the tasks once more to check the validity of task analysis findings, and to generate innovative ideas for the user interfaces of our mobile application. The primary purpose of the workshop was to design the interfaces with the actual users' participation. Then, sketching, paper prototyping and wireframing methods were used. The subjects were informed briefly about the design methods used. At the end of the workshop, a low fidelity representation of the application was designed.

**Fig. 3:**
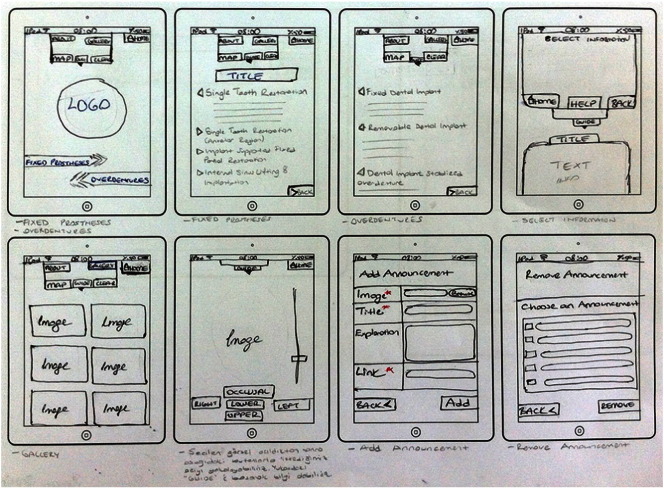
Sketching of possible approaches.

Sketches are instantiations of the design concepts which are used predominantly in the early ideation stages, to explore the huge range of design options, and dispose of ideas that do not fit specific design challenges [52]. It is an expendable technique and is fun [53]. Sketches do not have to be pretty, therefore the participants do not need to have any artistic ability but they should be able to explain them to others. We established an entertaining environment with the participants where the design ideas were discussed, critiqued, and realized for later reflection. Time limits from ten to thirty minutes were followed to encourage greater focus on producing diverse ideas rather than focusing on the details [54]. Final sketches worth developing were chosen for the following phase. A low-cost and fast pencil-paper sketch was prepared for the initial user interface design [55]. Fig. 3 presents the findings of sketching sessions. Sketching sessions were essential in the software planning and task clarification phases [56].

Although sketching and paper prototyping are similar tools as they are cheap, throwaway and fast, we continued with the paper prototyping in order to present the content on the sketched layouts. In this research, paper prototyping was a useful technique for working through the details of screen flows, sequencing, validating decisions about screen layout, button placements, and rough ideas for touch and gesture [57].

**Fig. 4:**
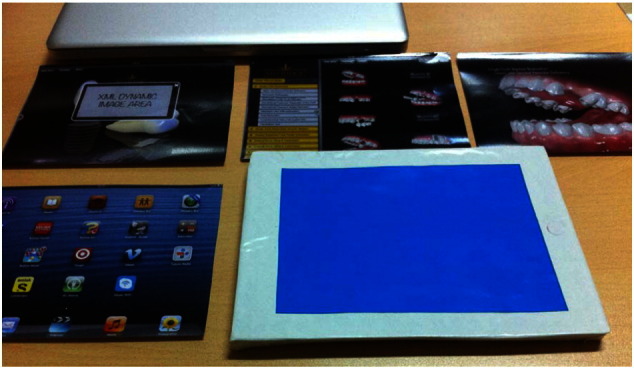
Sample of paper-prototyping.

The user interface layouts were detailed with visual components and colors to present the user experience on paper prototypes (Fig. 4). This is a tangible way of testing our interaction ideas with dentists and gathering feedback at an early stage in the design process [9]. User interfaces proposed by dentists were then presented and criticized by designers in the research team [48].

**Fig. 5:**
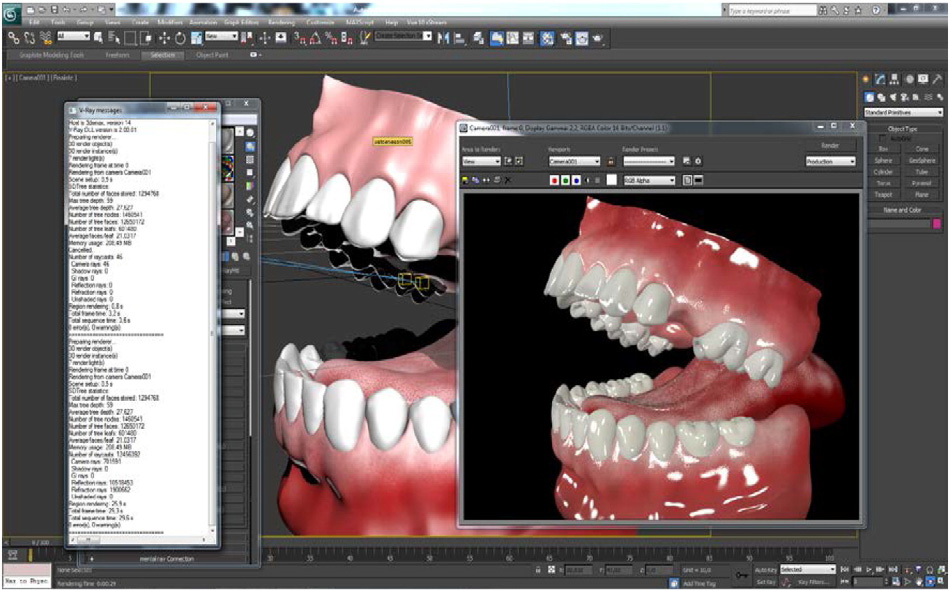
Content generation and creation.

**Fig. 6:**
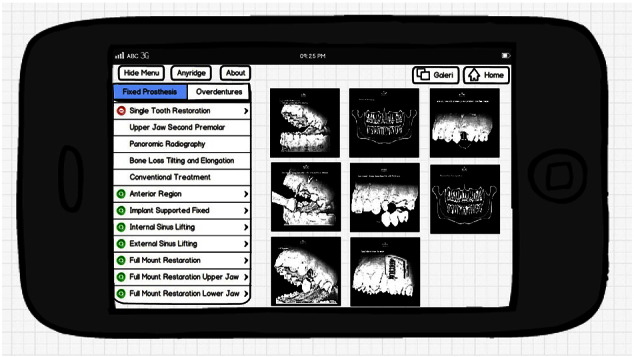
Wireframing.

Content generation and creation are shown in Fig. 5. A wireframe was prepared to show and check the structure, information hierarchy, functions, and content. Wireframes are a representation of the skeletal structure of a mobile application, compared to a building's blueprints [58]. Wireframes are used to lay out the structure, hierarchy and relationship between elements that make up a mobile application. They guide the user through a full experience without being distracted by the visual design. Well-designed wireframes provide a clear understanding of the structure and functionality of an application [59]. Functions, behaviors and content were reviewed using the low fidelity prototype (Fig. 6).

Balsamiq was selected to develop the mockup due to its agility and useful effects [60]. Notes were taken directly on sketches, wireframes, and sketch boards during critiques. The outcomes were all listed and applied in the final version of the application.

**Fig. 7:**
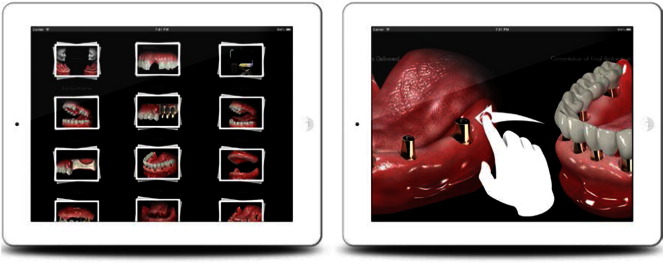
In-screen prototypes.

Screen layouts were designed using a photo editing tool and any necessary resizing of the design files was completed. The resized images were saved in a supported file format for the tablet devices. All screen images were organized into the correct order for the scenario (Fig. 7).

### 2.4 Implementation

Objective-C was selected as the programming language, Actionscript 3.0 for platform specific prototyping, and Extensible Markup Language (XML) was used for dynamic announcements. MySQL was used for the data collection. XCode 4.5 (4G182), Software Development Kit (SDK), Altova XMLSpy 2011 for XML, iOS, and Debugger: LLDB were used as IDE and tools in order to develop the mobile application.

The application needed to be practical and easy enough to be used by the dentists regardless of their mobile computing experience and skills. We assumed that users already possessed simple knowledge of finger taps and gestures used in touch-screen devices. When dentists click on a button corresponding events should be performed promptly. The application size should not exceed 200 MB including images, audio and all other application data. The application was designed to take no more than three steps or be more than three screens deep when using any feature [48].

The Model-View-Controller Pattern (MVC) was used to break the complex application into smaller parts to simplify the process. Also, MVC helps ensure maximum reusability. A split view controller was used in our application as it is the most common tablet device view controller. This is very effective for browsing through content and is a great starting point for content-heavy tablet device applications.

A hierarchical menu structure was designed and the tasks were categorized into different groups based on their similarities. The primary navigation was located on the left hand side of the screen, and the dynamic content was positioned at the center.

In order to check the possibilities of crash or error cases, the application had to be imported on to a real tablet device. The application initially demonstrated certain execution complications such as blank announcement windows, unexpected ineffective performances in terms of physical resources consumption and alignment. Detected functional and display related failures were removed.

### 2.5 Final product

The primary user target of our application is the dentists. We have identified two main categories labeled as “Fixed Prostheses” including nine items and “Overdentures” including four options. All tasks associated with these main categories are listed in Tables A.1 and A.2 in Appendix A.

**Fig. 8:**
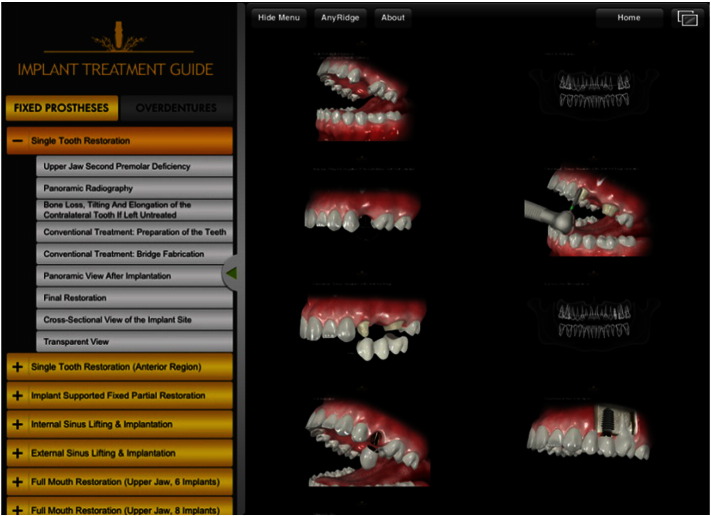
Final design.

Fig. 8 presents the final design of the application. A hierarchical menu structure was implemented. The screens were designed to have one function per screen to reduce the mental load [61]. The application provides flexibility to the users while also allowing navigation without any constraints.

**Fig. 9:**
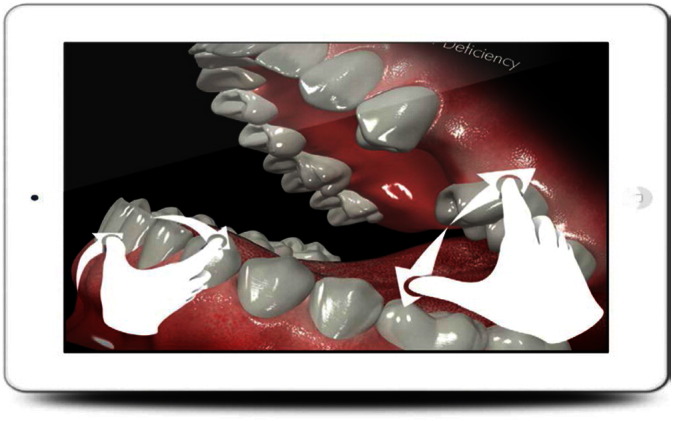
Content screen while pinching and rotating.

The interaction with the content screens is performed by pinching and rotating the screen as shown in Fig. 9. This allows users to move the 3D illustrative content in any direction to improve the visibility and functionality.

An iPad or newer with iOS 5.0 or higher versions operating system is the minimum hardware configuration to run the application at the satisfactory level. Wi-Fi or cellular network connection might be required in order to display the updated announcements. Minimum space required is 170 MB.

## 3 Evaluation

**Fig. 10:**
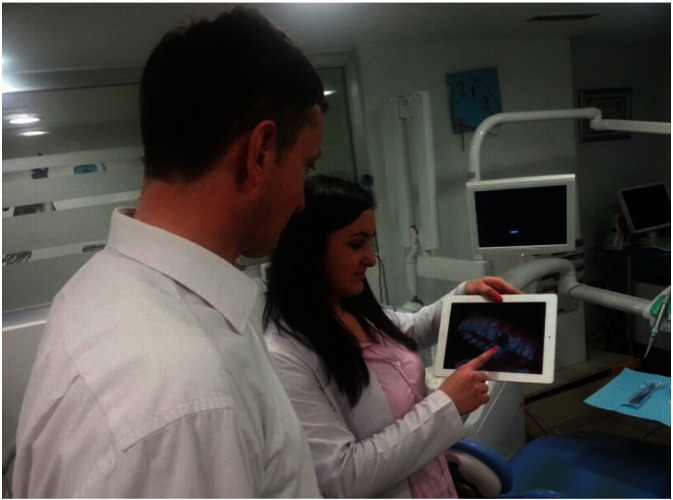
On-site patient–dentist interaction during evaluation.

The application was evaluated in terms of PEU, and PU using an online survey. A 7-point Likert scale was used. The survey consists of three parts: Part I is for collecting the demographic information of participants; Part II is for evaluating the PEU; and Part III is for measuring the PU. The online survey was accessible for a period of 25 days. Evidence that shows the user continuation of the application was determined by a semi-structured interview via phone. Fig. 10 shows the work-site use of the application by participating dentists.

We contacted the participants on the first day after he/she had downloaded the application to provide brief information about the experiment. The participants tried operating the application in their natural settings. We kept collecting updates from the subjects to clarify whether they were continuing to use our application for their business purposes. The dentists were encouraged to use the application to inform the patients in implant dentistry cases for treatment decision-making purposes.

### 3.1 Participants

The participants were dentists, categorized based on gender, age, years of experience as a professional, and amount of tablet device usage. 114 data sets were collected and 100 (24 female, and 76 male) were valid. Subjects declared their familiarity with tablet devices and mobile applications. Table 1 shows the details about the participants.

## 4 Evaluation results

### 4.1 Perceived ease. of use (PEU)

The Cronbach's alpha value of PEU is 0.82 which leads to a consistent and reliable set of data. The mean value of the collected data on PEU is 4.93 (STD = 0.12). The mean values are 4.66 (STD = 1.27) and 5.77 (STD = 0.96) respectively for male and female participants. It was found that there is significant difference between genders on PEU (t (98) = − 3.947, p = 0.000). Participants who were aged between 25 and 34 had the greatest mean value (M = 5.69, STD = 1.18). ANOVA was implemented to find out the significance between age groups. TUKEY test was also conducted for further analysis in understanding the differentiations among dentist's age range variable. Significant differences were found between age ranges (F = 5.54, p < 0.05). Especially participants aged between 25 and 34 felt that our application was easy to use.

ANOVA showed that job experience presented significant difference between identified categories on PEU (F = 6.51, p < 0.05). Specifically, dentists who have professional experience of between 5 and 9 years found the application easy to use.

It was also found that there is a significant difference between examined groups in terms of tablet device usage extent (F = 3.21, p < 0.05). There are significant differences especially between dentists who have been using tablet devices for less than 5 months and those with over 26 months of experience.

### 4.2 Perceived usefulness (PU)

The Cronbach's alpha value of PU is 0.86. We also have a consistent and reliable set of data in measuring PU. The mean value of PU is 5.22 (STD = 0.11). The results regarding user experience on PU are satisfactory. The mean values are 5.89 (STD = 0.96) and 5.01 (STD = 1.12) for male and females respectively. Female dentists found the application to be more useful than the male dentists (t (98) = − 3.493, p = 0.001).

It was found by ANOVA that age has a significant effect on PU (F = 3.28, p < 0.05). The TUKEY test was applied to find out the differentiation among dentists' age ranges.

ANOVA reported that working experience has a significant effect on PU (F = 3.04, p < 0.05). TUKEY results showed that dentists who have professional experience of between 5 and 9 years, and those whose experience is over 20 years are significantly different (p < 0.05).

ANOVA revealed that the extent of tablet device usage has a significant effect on the usefulness (F = 4.46, p < 0.05). The length of experience with mobile devices is correlated with the perceived usefulness.

We have found significant evidence that users would continue to use the application. 89 of the participants who responded to the survey question “Would you want to use the application again?” said that they would, often very decisively. Dentists aged relatively older generated a resistance to a change for their applications on medical procedures. A positive initial experience with the application encourages users to continue using it. We believe to report upon the development and evaluation of the system in a later research.

### 4.3 Subjective responses

At the end of the survey, a short semi-structured interview via phone was carried out to explore user reactions to the system. Responses indicated a strong preference for the use of our application by dentists. Additionally, respondents clearly would have continued to use the system: all participants who replied to the question “Would you continue to use the application?” (n = 100) mentioned that they would, very conclusively. Participants also expressed that they felt more comfortable and satisfying while explaining the treatment plan to the patients by the illustrations instead of presenting the bloody real case photographs: “The application saves time and effort. It removes the communication barriers between the dentist and the patients. I would definitely continue using it”. Another subject indicated that the application also provides greater control of their workflow, and improves the quality of service.

In this research, our main concern was designing and developing the mobile application with the actual users' participation. As further, we hope to extend the content of the application and report details on the development and usability evaluation of the system by different methodologies.

## 5 Discussions

This paper suggests that it is appropriate to design mobile systems for patient education and communication through the implementation of interactive technologies. An effective interaction between dentists and patients is the core of successful dentistry. The treatment decision making should be managed together and the patient has the right to be informed in detail. The portability of new generation devices provides flexibility to dentists in effectively communicating with the patients. Previous studies showed that mobile applications, as supportive learning tools for patient education, are a great way to educate patients and provide a visual guide when dentists are explaining their treatment procedures [29], [30], [31], [33].

This study differs from similar research in the literature. Related studies concentrated on the impact of clinical office computing to improve the interaction between physicians and patients while dealing with chronic diseases [62], [63], [64]. Few have focused on computerized systems in a dental environment and these studies do not present a mobile application [65], [66]. However, our mobile system was developed specifically for implant procedures by implementing user-centered design methodology to improve patient education and communication.

Dentists stated that the application saves time and that it reduces the required effort. It also provides dentists greater control of their workflow, and improves the quality of service. The rich visual components support the user experience and interaction. Participants indicated that they are willing to continue using this application in the future. It was revealed that the designed menu structure, and 3D illustrative content appear to be user friendly for dentists and more attractive for patients on a visual impact basis. Therefore, it is more likely to produce better results compared with previously used text-based and bloody presentations taken from real cases.

Patients trust businesses which are leaders in the field and offering patients a mobile application shows that the dentist cares enough to provide a high quality service through technological investments. The adoption of mobile applications in dentistry for educative purposes continues to progress and become more important as an alternative to the traditional communication methods. Statistical results show that the application was adopted successfully by the dentists. The flexibility of interaction provided by the touchscreen might encourage the users to adopt the system more rapidly in today's information age. Dentists who are more familiar with tablet devices declared that the application is very effective in explaining the treatment plan to patients.

The main strength of this research is in the designing of the application with the participation of actual users. Implemented methodologies in application development life cycle are encouraging for the further mobile development projects and participants who believe the application is more familiar and usable when their preferences are included. Participants also believe that the use of advanced technologies in their workflow would improve the quality of service. They are much more encouraged to use mobile devices for business purposes. Our application improves the information sharing behavior of dentists to enhance the patients' right to make informed decisions. Designers of mobile information systems need to be aware of the importance of the user interface in dentist–patient communication.

## 6 Conclusion

In conclusion, user centered development methodology was successfully implemented to develop a mobile dental application. The needs and preferences of the dentists were identified during on-site visits. To understand the regular workflow, the dentists were observed and interviewed in their professional environments. The involvement of the end-users allowed us to generate better design solutions, and develop a more practical and usable application. Dentists believe that this approach produces better designs, which is helpful in promoting interaction for all stakeholders. The application advances the information sharing behavior of dentists to enhance the patients' right to make informed decisions. It is strongly believed that the modularity of the design approach and software architecture used in this study is beneficial for further related applications in medical informatics. As a further research, the application is intended to be evaluated by scientifically approved usability inspection methods and the findings can be compared with a commercial equivalent.

## Figures and Tables

**Table A.1:** Fixed prostheses menu.

Menu item	Subcategories
1.1. Single tooth restoration	1.1.1. Upper jaw second premolar deficiency
	1.1.2. Panoramic radiography
	1.1.3. Bone loss, tilting and elongation of the contralateral tooth
	1.1.4. Conventional treatment: preparation of the teeth
	1.1.5. Conventional treatment: bridge fabrication
	1.1.6. Panoramic view after implantation
	1.1.7. Final restoration
	1.1.8. Cross-sectional view of the implant site
	1.1.9. Transparent view
1.2. Single tooth restoration (anterior region)	1.2.1. Upper jaw central incisor deficiency
	1.2.2. Panoramic radiology — before
	1.2.3. Bone loss over time, irreversible unaesthetic results
	1.2.4. Bone loss over time panoramic view
	1.2.5. Conventional treatment: preparation of the teeth
	1.2.6. Conventional treatment: bridge fabrication
	1.2.7. Panoramic view after implantation
	1.2.8. Titanium angled abutment/porcelain-fused-to-metal crown
	1.2.9. Final restoration and cross-sectional view of the bone
	1.2.10. Zirconium abutment
	1.2.11. Final restoration and cross-sectional view of the bone
1.3. Implant supported fixed partial restoration	1.3.1. Upper jaw 2nd premolar, 1st & 2nd molar deficiency
	1.3.2. Panoramic radiography — before
	1.3.3. Conventional treatment: removable partial denture
	1.3.4. Deformation due to partial denture
	1.3.5. Bone loss
	1.3.6. Pneumatization of maxillary sinus
	1.3.7. Implantation
	1.3.8. Panoramic view
	1.3.9. Cross-sectional view of the implant
	1.3.10. Cross-sectional view, close-up
	1.3.11. Final Restoration, Transparent View
1.4. Internal sinus lifting & implantation	1.4.1. Upper jaw 2nd premolar, 1st & 2nd molar deficiency
	1.4.2. Pneumatization of maxillary sinus
	1.4.3. Panoramic view
	1.4.4. Panoramic views after sinus lifting & implantation
	1.4.5. Sinus lifting, grafting & implantation
	1.4.6. Abutments delivered
	1.4.7. Panoramic views after final prosthesis
	1.4.8. Cross-sectional view of implants
	1.4.9. Transparent view
1.5. External sinus lifting & implantation	1.5.1. Upper jaw 2nd premolar, 1st & 2nd molar deficiency
	1.5.2. Severe pneumatization of maxillary sinus
	1.5.3. Panoramic view
	1.5.4. Panoramic views after sinus lifting & implantation
	1.5.5. Sinus lifting, grafting & implantation
	1.5.6. Abutments delivered
	1.5.7. Panoramic views after final prosthesis
	1.5.8. Cross-sectional view of implants
	1.5.9. Transparent view
1.6. Full mouth reconstruction (upper jaw, 6 implants)	1.6.1. Loss of vertical dimensions
	1.6.2. Edentulous upper & lower jaws
	1.6.3. Panoramic view of edentulous upper & lower jaws
	1.6.4. Edentulous upper jaw, 6 implants
	1.6.5. Following osteointegration abutments delivered
	1.6.6. Cementation of final restoration
	1.6.7. Transparent view
	1.6.8. Final restoration
	1.6.9. Panoramic view
1.7. Full mouth reconstruction (upper jaw, 8 implants)	1.7.1. Loss of vertical dimension
	1.7.2. Edentulous upper & lower jaws
	1.7.3. Panoramic view of edentulous upper & lower jaws
	1.7.4. Edentulous upper jaw, 8 implants
	1.7.5. Following osteointegration abutments delivered
	1.7.6. Abutments delivered, occlusal view
	1.7.7. Transparent view
	1.7.8. Final restoration
	1.7.9. Panoramic view
1.8. Full mouth reconstruction (lower jaw, 6 implants)	1.8.1. Loss of vertical dimension
	1.8.2. Edentulous upper & lower jaws
	1.8.3. Panoramic view of edentulous upper & lower jaws
	1.8.4. Edentulous lower jaw, 6 implants
	1.8.5. Following osteointegration abutments delivered
	1.8.6. Cementation of final restoration
	1.8.7. Final restoration transparent view
	1.8.8. Panoramic view
1.9. Full mouth reconstruction (lower jaw, 8 implants)	1.9.1. Loss of vertical dimension
	1.9.2. Edentulous upper & lower jaws
	1.9.3. Panoramic view of edentulous upper & lower jaws
	1.9.4. Edentulous lower jaw, 8 implants
	1.9.5. Following osteointegration abutments delivered
	1.9.6. Cementation of final restoration
	1.9.7. Final restoration transparent view
	1.9.8. Panoramic view

**Table A.2:** Overdenture main category items.

Menu item	Subcategories
2.1. Single tooth restoration	2.1.1. Loss of teeth & bone
	2.1.2. Totally edentulous mandible
	2.1.3. Panoramic view
	2.1.4. Delivery of octa abutments
	2.1.5. Delivery of bar construction
	2.1.6. Bar construction fixed by screw
	2.1.7. Attachments
	2.1.8. Overdenture cross-section
	2.1.9. Delivery of overdenture
	2.1.10. Transparent view
2.2. Single tooth restoration (anterior region)	2.2.1. Loss of teeth & bone
	2.2.2. Totally edentulous mandible
	2.2.3. Panoramic view
	2.2.4. Delivery of octa abutments
	2.2.5. Delivery of bar construction
	2.2.6. Transparent view
	2.2.7. Overdenture reinforcement with metal construction
	2.2.8. Overdenture cross-section
2.3. Implant support fixed partial restoration	2.3.1. Loss of teeth & bone
	2.3.2. Totally edentulous mandible
	2.3.3. Panoramic view
	2.3.4. Delivery of ball abutment
	2.3.5. Delivery of attachments
	2.3.6. Cross-section
	2.3.7. Transparent view
2.4. Internal sinus lifting & implantation	2.4.1. Loss of teeth & bone
	2.4.2. Totally edentulous mandible
	2.4.3. Panoramic view
	2.4.4. Delivery of kerators
	2.4.5. Transparent view
	2.4.6. Delivery of attachments
	2.4.7. Cross-section of attachments
	2.4.8. Final restoration
	2.4.9. Transparent view

**Table 1:** Information on participants.

Variables	Categories	Frequency	Percentage
Gender	Female	24	24
	Male	76	76
Age range	− 24	18	18
	25–34	31	31
	35–44	24	24
	45–54	20	20
	55 +	7	7
Dentistry professional experience (in years)	− 4	32	32
	5–9	18	18
	10–14	16	16
	15–19	16	16
	20 +	18	18
Duration of tablet device usage (in months)	− 5	33	33
	6–11	11	11
	12–17	13	13
	18–25	14	14
	26 +	29	29
